# Accelerating COVID-19 Vaccination Among People Living With HIV and Health Care Workers in Tanzania: A Case Study

**DOI:** 10.9745/GHSP-D-23-00281

**Published:** 2024-06-27

**Authors:** Mohamed F. Jalloh, Florian Tinuga, Mohamed Dahoma, Anath Rwebembera, Ntuli A. Kapologwe, Daniel Magesa, Kokuhabwa Mukurasi, Oscar Ernest Rwabiyago, Jaiving Kazitanga, Angela Miller, David Sando, Haruka Maruyama, Redempta Mbatia, Florence Temu, Eva Matiko, Kokuhumbya Kazaura, Prosper Njau, Jennifer Imaa, Tara Pinto, Sophia A. Nur, Nicolas Schaad, Augustine Malero, Damian Damian, Jonathan Grund, George S. Mgomella, Alison Johnson, Gbolahan Cole, Eunice Mmari, Wangeci Gatei, Mahesh Swaminathan

**Affiliations:** a Tanzania Country Office, U.S. Centers for Disease Control and Prevention, Dar es Salaam, Tanzania.; b Immunization and Vaccine Development, Tanzania Ministry of Health, Dar es Salaam, Tanzania.; c Zanzibar Integrated HIV, Hepatitis, TB and Leprosy Program, Zanzibar, Tanzania.; d National AIDS Control Programme, Tanzania Ministry of Health, Dodoma, Tanzania.; e President’s Office - Regional Administration and Local Government, Dodoma, Tanzania.; f Management and Development for Health, Dar es Salaam, Tanzania.; g Tanzania Country Office, ICAP at Columbia University, Dar es Salaam, Tanzania.; h Tanzania Health Promotion Support, Dar es Salaam, Tanzania.; i Tanzania Country Office, Amref Health Africa, Dar es Salaam, Tanzania.

## Abstract

**Background:**

There is limited evidence on COVID-19 vaccination uptake among people living with HIV (PLHIV) and health care workers (HCWs), with the current evidence concentrated in high-income countries. There is also limited documentation in the published literature regarding the feasibility and lessons from implementing targeted vaccination strategies to reach PLHIV and HCWs in low- and middle-income countries.

**Program Development, Piloting, and Implementation:**

We designed and implemented multifaceted strategies to scale up targeted COVID-19 vaccination among PLHIV and HCWs in 11 administrative regions on the mainland of Tanzania plus Zanzibar. An initial 6-week intensification strategy was implemented using a diverse partnership model comprising key stakeholders at the national- and subnational levels. A layered package of strategies included expanding the number of certified vaccinators, creating vaccination points within HIV clinics, engaging HCWs to address their concerns, and building the capacity of HCWs as “champions” to promote and facilitate vaccination. We then closely monitored COVID-19 vaccination uptake in 562 high-volume HIV clinics. Between September 2021 and September 2022, the proportion of fully vaccinated adult PLHIV increased from <1% to 97% and fully vaccinated HCWs increased from 23% to 80%.

**Lessons and implications:**

Our intra-action review highlighted the importance of leveraging a strong foundation of existing partnerships and platforms, integrating COVID-19 vaccination points within HIV clinics, and refining strategies to increase vaccination demand while ensuring continuity of vaccine supply to meet the increased demand. Lessons from Tanzania can inform targeted vaccination of vulnerable groups in future health emergencies.

## Abstract (precis)

Within a 12-month period, targeted strategies increased COVID-19 vaccination uptake, minimized wastage of limited vaccine supply, and reduced missed opportunities for vaccination among people living with HIV and health care workers in Tanzania.

## Teaser

**Key Messages:**

- Quickly leveraging an existing partnership model for HIV health services was essential in scaling up COVID-19 vaccination uptake among people living with HIV (PLHIV) and health care workers (HCWs) in Tanzania.
- Engaging HCWs to address their COVID-19 vaccination concerns and increasing their vaccination uptake was a critical first step to improving vaccination uptake among PLHIV who may have been hesitant to get vaccinated.
- Creating COVID-19 vaccination points within HIV clinics made it convenient for PLHIV, their families, and people in their networks to get vaccinated, which likely helped to increase uptake and reduce missed opportunities for vaccination.

**Key Implications:**

- Our experience suggests that integrating vaccination promotion and vaccination service delivery into a unified intervention delivered by credible HCWs is a promising approach for scaling up vaccination uptake in high-risk populations during health emergencies.
- Empowering HCWs to make certain localized decisions during emergency response efforts could result in simple and context-appropriate innovations, as observed when HCWs avoided vaccine wastage within their health facilities in Tanzania.

## BACKGROUND

People living with HIV (PLHIV) who are immunocompromised may be at increased risk of severe illness and death from COVID-19.^1^^–^^3^ Several studies demonstrated the safety and immunogenicity of COVID-19 vaccines among PLHIV.^4^^–^^6^ The World Health Organization (WHO) recommends COVID-19 vaccination for PLHIV.^7^ However, little is known about the progress toward vaccinating PLHIV outside of high-income countries. Low-income countries experienced limited supply of COVID-19 vaccines and reported lower coverage of COVID-19 vaccination compared to wealthier countries.^8^ At the same time, population-based studies revealed high seroprevalence of SARS-CoV-2 in sub-Saharan African countries.^9^ A WHO study found that two-thirds of people living in sub-Saharan Africa have been infected with SARS-CoV-2.^10^

Current evidence on vaccination uptake among PLHIV mostly comes from high-income countries. In the United States, less than 6 months after COVID-19 vaccines became available, 64% of a national sample of PLHIV had received at least 1 dose of a COVID-19 vaccine.^11^ Other studies have also examined the willingness of PLHIV to accept COVID-19 vaccination but without determining their vaccination uptake. A cross-sectional study in Wuhan, China, found that 61% of PLHIV were willing to get vaccinated against COVID-19 compared to 81% who were willing among the general population.^12^ There is also limited documentation in the literature regarding the feasibility and lessons from implementing targeted vaccination strategies to reach PLHIV and other groups at higher risk of severe COVID-19 disease.

There is limited documentation in the literature regarding the feasibility and lessons from implementing targeted vaccination strategies to reach PLHIV and other groups at higher risk of severe COVID-19 disease.

Tanzania received its first shipment of COVID-19 vaccines in July 2021 and began vaccinating an age-eligible population of adults aged 18 years and older in August 2021.^13^ A total of 1,058,400 doses of Janssen COVID-19 vaccine were initially made available to Tanzania through COVAX Facility coordinated by WHO as part of a donation from the U.S. Government. Other COVID-19 vaccine products were later introduced by the Government of Tanzania, including those requiring 2-dose primary series. Although the public’s initial demand for the single-dose Janssen COVID-19 vaccine was high in the first few weeks of becoming accessible, the number of weekly vaccine doses administered plummeted quickly. The slowdown in vaccination appeared to have been influenced by similar hindering factors observed elsewhere, such as operational challenges in vaccine delivery, limited preexisting infrastructure for adult vaccination, and ubiquity of misinformation that derailed confidence and trust in the vaccines.^14^^–^^16^

In this program case study, we describe our joint design and scale-up of COVID-19 vaccination strategies to reach PLHIV and health care workers (HCWs) in 11 regions on the mainland of Tanzania plus Zanzibar. We then quantify key programmatic achievements, provide illustrative vaccine uptake data from routine reporting among adult PLHIV in high-volume facilities, and further reflect on key lessons learned and challenges encountered. Our lessons from Tanzania may inform targeted vaccination elsewhere among populations at higher risk of severe disease in future health emergencies where vaccination becomes a countermeasure component of the public health response.

## STRATEGIES TO SCALE UP COVID-19 VACCINATION AMONG PEOPLE LIVING WITH HIV AND HEALTH CARE WORKERS

An initial 6-week intensification effort to reach PLHIV and HCWs was implemented between September 27 and November 7, 2021. The intensification effort was implemented using a diverse partnership model among key stakeholders at the national- and subnational levels. The initial intensification focused on high-volume HIV clinics to: (1) expand the number of certified vaccinators, (2) create vaccination points in high-volume HIV clinics starting with administrative regions where there was strong buy-in from regional health authorities (e.g., regional medical officers and regional commissioners), (3) engage HCWs to address their concerns and build their vaccination confidence, (4) strengthen capacity of HCWs and community health workers to facilitate vaccination in facility- and community-based settings, and (5) monitor uptake of COVID-19 vaccination in high-volume facilities. Core strategies and activities from the initial intensification were expanded jointly through close collaboration with the regional health authorities, the national immunization program, and the national HIV program in Tanzania. COVID-19 vaccination activities were integrated into routine joint supportive supervision to provide real-time feedback and guidance to an expanded set of health facilities.

### Expanding the Number of Certified Vaccinators

Leveraging the existing government-approved training for COVID-19 vaccination, over 3,000 HCWs were swiftly trained to become approved COVID-19 vaccinators. Expanding the number of certified vaccinators was a foundational step that subsequently allowed for the integration of fixed sites for COVID-19 vaccination within HIV clinics. Beyond the HIV clinics, HCWs in other parts of the health facilities, such as outpatient departments, also benefited from the trainings and became certified. The expanded and integrated training approach enabled the eventual expansion of COVID-19 vaccination fixed sites in multiple locations throughout health facilities, including outpatient departments. These synergistic training approaches for vaccinators from the initial intensification efforts provided a longer-term platform for refresher trainings when new COVID-19 vaccine products were added to the stock. We learned from this experience that targeting HCWs in HIV clinics provided a cost-effective entry point for subsequent expansion of COVID-19 vaccination efforts at the facility and community levels.

### Creating Vaccination Points in HIV Clinics

Convenience is an important factor in enabling people to get vaccinated.^17^ Bringing vaccination services closer to populations in need has shown to be an effective strategy to increase vaccination uptake.^18^ Fixed COVID-19 vaccination points were created in HIV clinics through close collaboration among implementing partners, local government health authorities, the Immunization and Vaccine Development Program, and the National AIDS Control Program. Implementing partners worked with the facility in-charges (the most senior official responsible for health services in a designated health facility) and immunization staff at the district, regional, and central levels to coordinate vaccine supply and ensure appropriate levels of vaccine stock in the HIV clinics. We documented that having multiple vaccination points in the facility made it more convenient to get vaccinated and helped reduce wastage of multidose vials. For example, there were instances when opened vials were transported between sites in the facility to minimize wastage as and when appropriate, following localized protocols and procedures. The HCWs at different vaccination points within the same health facilities communicated with each other when they had open vials but not sufficient people present to vaccinate. Open vials were transported in small cold boxes to other vaccination points within the facility to prevent wastage. There was no standardized national or regional protocol for doing so, but HCWs came up with local simple innovations on their own to communicate and coordinate with each other. An important lesson learned is that empowering HCWs to make decisions locally can result in simple and context-appropriate innovations, as observed with local measures taken to avoid vaccine wastage in Tanzania.

The additional vaccination points in HIV clinics were not restricted to PLHIV and were appropriately leveraged to expand vaccination access, including for unvaccinated HCWs, family members of PLHIV, and others in the networks of PLHIV more broadly. We observed that the creation of COVID-19 vaccination points in HIV clinics became a best practice that was quickly replicated across all regions in Tanzania, which helped to boost nationwide uptake of COVID-19 vaccination among PLHIV, their families, and networks.

The additional vaccination points in HIV clinics were not restricted to PLHIV and were appropriately leveraged to expand vaccination access, including for unvaccinated HCWs, family members of PLHIV, and others in the networks of PLHIV more broadly.

### Engaging Health Care Workers and Addressing Their Concerns

The integration model leveraged teams of locally employed personnel who were already providing HIV-related supportive supervision in HIV clinics to do the same for COVID-19 vaccination. The teams were trained on evidence-based strategies to build vaccine confidence among HCWs (Supplement). This follow-up training was prompted by the low COVID-19 vaccination uptake observed among HCWs within the first 6 months of the vaccines becoming available in Tanzania. The trained team members, working in collaboration with local health authorities and facility in-charges, used a training-of-trainers approach to meet individually with HCWs on COVID-19 vaccination to strengthen their confidence in vaccination, enable them to become active promoters of vaccination, and help them mentor other HCWs on building vaccine confidence. This was a critical strategy given that unvaccinated HCWs were not viewed as trusted communicators to promote COVID-19 vaccination. A critical lesson from this experience reinforces the need to prioritize vaccinating HCWs early so that they can be effective promoters to strengthen vaccination confidence among their clients and community members at large.

### Building Capacity to Facilitate Vaccination of People Living With HIV

During scheduled HIV clinic visits, HCWs were trained to determine the COVID-19 vaccination status of PLHIV. Unvaccinated PLHIV were counseled by HCWs and encouraged to be vaccinated. Those who agreed were vaccinated onsite at the HIV clinics.

Together with the facility in-charges, the implementing partners used a call-back strategy in which they called PLHIV who received multimonth dispensing (MMD) of antiretroviral drugs. In some facilities, in consultation with regional stakeholders, minimal transportation costs for those on MMD were reimbursed, given that they were being asked to make an extra visit to the HIV clinic outside of their scheduled visit. The call-back approach facilitated the vaccination of PLHIV on MMD and likely helped to reduce vaccination inequities among this subpopulation of PLHIV. In addition, health facilities were supported to disseminate COVID-19 vaccine educational materials in high-traffic areas to encourage patients and their families to get vaccinated. Moreover, community health workers and expert clients were trained to support the integration of COVID-19 vaccination promotion into preexisting HIV-related outreach. Community activities included addressing misinformation about vaccination and engaging PLHIV-led community-based organizations at the grassroots level. We learned that as the vaccines are being promoted, they should also made available at the same time to avoid missed opportunities for vaccination. In essence, vaccination demand and vaccine delivery efforts are optimized when they are integrated into a unified intervention bridged by HCWs.

### Monitoring Uptake of COVID-19 Vaccination

A weekly aggregated Excel-based uptake monitoring system was rapidly implemented and launched initially in 124 high-volume HIV clinics by the end of September 2021. The Excel template was periodically modified and scaled up to include all 562 high-volume HIV clinics supported by the U.S. Centers for Disease Control and Prevention (CDC) in Tanzania and Zanzibar by December 2021. At the end of each week, implementing partners reported the aggregated number of PLHIV and HCWs in their supported sites who were fully vaccinated (i.e., received the recommended primary series doses for the specified vaccine product), partially vaccinated (i.e., missed 1 of the primary series doses of a multidose vaccine), unvaccinated (i.e., had not received any COVID-19 vaccine dose), or had unknown vaccination status. Adult PLHIV were defined as diagnosed people living with HIV who are aged 20 years or older and enrolled in HIV care and treatment services in a health facility supported by CDC in 11 regions on Tanzania mainland and Zanzibar. This age cut-off was used because it had existing reliable denominators across the supported HIV clinics that fed into the weekly monitoring of the proportion of PLHIV. HCWs included all clinical staff, administrative personnel, and other nonclinical staff in health facilities supported by CDC in 11 regions on the mainland of Tanzania and Zanzibar. Between September 2021 and September 2022, the proportion of fully vaccinated adult PLHIV increased from <1% to 97% and the proportion of fully vaccinated HCWs increased from 23% to 80% in the monitored facilities (Figure).

No data system existed for monitoring COVID-19 vaccination uptake among PLHIV and HCWs at the onset of the scale-up efforts. The rapidly deployed aggregated reporting system had key benefits. First, it allowed us to have weekly snapshots of the progress that implementing partners were making with vaccinating PLHIV and HCWs against targets that were assigned to them. Weekly reporting helped ensure accountability of vaccination resources and informed continuous programmatic adjustments. For example, enhanced site support, including supportive supervision visits, was undertaken in low-performing sites. Teams visited high-performing sites and regions to observe best practices that were later shared with other sites and regions to improve overall performance. Weekly data review meetings were conducted with the implementing partners to discuss successes and jointly come up with adaptive strategies to address emerging challenges.

Rapid deployment of an aggregated reporting system enabled monitoring of vaccination progress, accountability of resources, and continuous programmatic adjustments.

The weekly aggregated reporting system also encountered several challenges. Although the underlying data reported by implementing partners were coming from the official vaccination registers in the health facilities, the vaccination data for PLHIV were not centrally aggregated nationally across all HIV clinics. This issue was initially attempted to be resolved by integrating new data fields within the Chanjo COVID national electronic immunization registry to indicate COVID-19 high-risk status of vaccinated people, including HIV status and other high-risk health conditions. This approach was severely limited by incomplete data on high-risk status in the Chanjo COVID system. Therefore, as a longer-term solution, a COVID-19 vaccination module was later developed by the Government of Tanzania and integrated into the patient-level data system (CTC2) that was already being used for clinical management of PLHIV nationally.

CTC2 is a patient-level registry owned and operated by the Government of Tanzania. It holds detailed information on HIV and TB/HIV patients. Data from CTC2 are aggregated monthly and fed into monthly reporting in the national DHIS2 platform. Chanjo COVID is an electronic patient registry developed by the Government of Tanzania specifically for managing COVID-19 vaccination records nationwide. Chanjo COVID is built upon the DHIS2 platform. Unfortunately, Chanjo COVID and CTC2 cannot directly connect and share data. To address this issue in the future, technological solutions are needed to enable electronic immunization registries like Chanjo COVID to connect with existing patient-level registries like CTC2. Connecting the 2 systems would result in more comprehensive patient-level data to inform clinical patient management and enable the evaluation of longitudinal patient outcomes. Additionally, the lack of national unique identifiers poses a challenge as it prevents the merging of patient-level HIV data with their COVID-19 vaccination data. This limitation reinforces the larger need for accelerating the use of unique identifiers nationally.

## INSIGHTS FROM INTRA-ACTION REVIEW

On July 15, 2022, an intra-action review with stakeholders was conducted to document lessons from scaling up COVID-19 vaccination to PLHIV, HCWs, and eventually the general population in CDC-supported regions in Tanzania. The workshop included participants from the Government of Tanzania, CDC, and the 4 implementing partners. Participants were divided into 6 thematic groups: (1) collaboration and coordination, (2) capacity-building, (3) data management, (4) demand creation, (5) supply chain, and (6) service delivery. The thematic groups deliberated and reported on successes, emerging improvements, and lessons learned for their respective areas.

Crosscutting themes that materialized from the group discussions included: (1) needing close partnerships and engagement of political, government, and religious leaders for planning, capacity-building, demand creation, and service delivery to enhance vaccination reach, uptake, and acceptance at community, regional, and national levels; (2) incorporating innovative strategies to address gaps in knowledge, supply chain, or service delivery, such as providing vaccination incentives, engaging peer champions, integrating with other health services, combining outreach and vaccination with mass community gatherings, and using live media coverage of vaccination and promotion; and (3) ensuring adequate preparation and planning to forecast vaccination demand, ensure timely data entry and management, and reduce vaccination wastage. The Table summarizes additional findings from the stakeholder workshop.

## EXPANDING INTO THE COMMUNITY

Following successes with scaling up COVID-19 vaccination among PLHIV and HCWs, the Government of Tanzania was supported to expand vaccination services to the general population. Enhanced vaccination strategies in this subsequent phase included the use of temporary fixed-vaccination sites in communities, deployment of mobile outreach teams in communities, and rapid integration of COVID-19 vaccination into other routine health services. The systems, workforce, and lessons learned from the initial efforts to vaccinate PLHIV and HCWs had “intentional spillovers” into vaccinating the general population. Transferrable skills in the trained workforce were constantly leveraged and repurposed to meet the evolving needs of the wider COVID-19 vaccination program. For example, the initially trained vaccinators through the targeted PLHIV vaccination efforts were later leveraged to support community-based vaccination activities. It is estimated that a total of 37.7 million people had been fully vaccinated in Tanzania by the end of December 2022. This number represents approximately 62% of the total population in Tanzania—the second-highest COVID-19 vaccination rate in sub-Saharan Africa after Rwanda.^19^

The systems, workforce, and lessons learned from the initial efforts to vaccinate PLHIV and HCWs had “intentional spillovers” into vaccinating the general population.

## COMPARATIVE PROGRESS AND CONSIDERATIONS

COVID-19 vaccines became accessible around early January 2021, but initial access was mostly restricted to high-income countries. In a multicountry cohort of PLHIV, 55% had received at least 1 dose of a COVID-19 vaccine by July 2021, with better uptake in high-income countries (71%) compared to Latin America and the Caribbean (59%), South Asia (49%), Southeast Asia (41%), and sub-Saharan Africa (18%).^20^ A study in New York City found that 63% of PLHIV had received at least 1 dose of a COVID-19 vaccine by October 2021.^21^ A separate state-wide study in the state of Oregon, United States, revealed that 62% of PLHIV in the state had received at least 1 dose of a COVID-19 vaccine as of June 2021.^22^ COVID-19 vaccination uptake among PLHIV has been reported to be as high as 86% in Turin, Italy, based on a study conducted between November 2021 and April 2022.^23^ Nevertheless, it is difficult to compare PLHIV vaccination uptake across studies because of varying methods in how the data have been captured from different data collection periods. COVID-19 vaccination data are almost nonexistent among subpopulations of PLHIV, including key populations (i.e., men who have sex with men, female sex workers, and people who inject drugs). In mainland China, only 9% of men who have sex with men who were living with HIV had received any COVID-19 vaccine as of April 2021.^12^

To our knowledge, no prior published study has provided nationally representative estimates of COVID-19 vaccination coverage among PLHIV. To address this evidence gap, the Tanzania HIV Impact Survey 2022–2023 incorporated a module that will provide nationally representative estimates of COVID-19 vaccination coverage among PLHIV in the country.^24^ Vaccination coverage among key populations cannot be ascertained through the survey, and routine data on COVID-19 vaccination uptake among key populations are not available in Tanzania. To further address this evidence gap, a module on COVID-19 vaccination was integrated into 2 planned bio-behavioral surveys among men who have sex with men, female sex workers, and people who inject drugs on Tanzania’s mainland and Zanzibar with data expected to be publicly available in 2025. Leveraging existing planned surveys with key and vulnerable populations to understand their COVID-19 uptake may inform more targeted outreach strategies to ensure vaccination equity for these groups.

Additional doses of COVID-19 vaccines are now largely accessible to the general population in many parts of the world but very rarely in most of sub-Saharan Africa.^25^ An immunogenicity study found a COVID-19 mRNA vaccine third dose to be tolerable among PLHIV and produced a strong humoral response in PLHIV.^26^ In fact, additional doses of COVID-19 vaccination among PLHIV may be required to provide robust protection against severe COVID-19,^4^ especially in the evolving context of SARS-CoV-2 variants of concern.^27^ For PLHIV with advanced HIV disease, WHO and other international regulatory bodies strongly recommend an additional dose that extends the primary series doses should be given 1–3 months after the primary series is completed.^28^ Targeted strategies used to reach PLHIV that we have highlighted in this article could be used to implement evidence-informed deployment strategies for additional doses of COVID-19 vaccines to PLHIV and other populations at greater risk for severe COVID-19 disease in Tanzania and elsewhere.

Going beyond COVID-19 vaccination, training HCWs in HIV clinics to vaccinate eligible adolescents with human papillomavirus (HPV) vaccination and coordinating the availability of the vaccines at the HIV clinics may help to boost HPV vaccination uptake among this population in the future. Although adult vaccination programs are limited in sub-Saharan Africa, similar approaches of integrating immunization services into other entry points and outpatient departments within health facilities can help to reach more people more conveniently with vaccines, including during future disease outbreaks where vaccines become an integral part of the public health response. Even when it may not be logistically feasible to directly integrate immunization services at alternate entry points (e.g., due to cold chain considerations), integrating vaccination screenings in multiple entry points within health facilities and making referrals to the fixed vaccination site can be done with minimal planning and without meaningfully additive costs.

## CONCLUSION

We have shown that targeting COVID-19 vaccines to groups that are at greater risk of severe COVID-19 disease is a promising strategy to increase vaccine uptake, minimize wastage of limited vaccine supply, and reduce missed opportunities for vaccination. Among many important factors of our program in Tanzania, relying on a partnership model that was built on existing health services delivery platforms was essential in scaling up vaccination uptake among PLHIV and HCWs in Tanzania. Creating a strong linkage between vaccine demand and supply-side factors was critical to increasing vaccination coverage among PLHIV, as exemplified by the creation of vaccination points in HIV clinics that leveraged trusted HCWs to simultaneously promote and deliver the vaccines. Equally important, we learned that empowering HCWs to make decisions resulted in simple and context-appropriate innovations, as observed with local measures taken by HCWs to avoid vaccine wastage within their health facilities. Our experience in Tanzania can inform targeted vaccination of vulnerable groups in similar settings with strong considerations for equity, integrated programming, and maximizing public health impact in the current COVID-19 pandemic and in future health emergencies where vaccines are part of the response.

**FIGURE:**
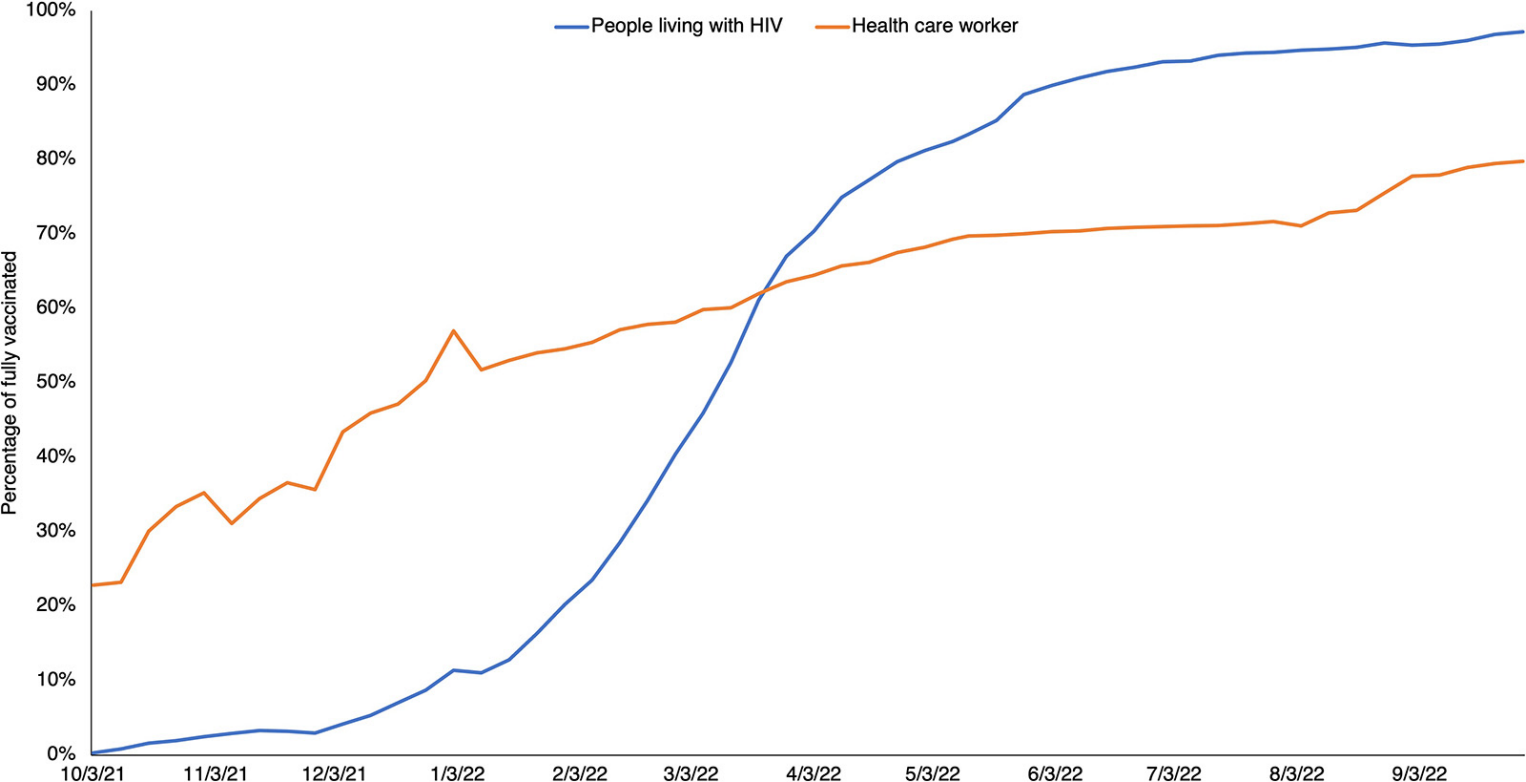
Trends in Percentage of PLHIV and HCWs Fully Vaccinated Against COVID-19 in Monitored Health Facilities, Mainland Tanzania and Zanzibar, October 2021–September 2022 Abbreviations: HCW, health care worker; PLHIV, people living with HIV.

**TABLE.** Successes, Emerging Improvements, and Lessons Learned From Thematic Groups at the Stakeholder Workshop on COVID-19 Vaccination Intensification and Scale-up

**Thematic area**	**Successes**	**Challenges**	**Lessons Learned**
Collaboration and coordination	Joint strategy and plan for COVID-19 vaccination uptake activities Formation of National Vaccine Pillar with diverse stakeholders Collaborative planning on developing microplans and implementation Political and government leadership commitment at regional level Timely availability and disbursement of funding	Unifying approaches took time Delay of microplan implementation at lower levels	Use bottom-up approaches to reduce time Enhance demand forecasting tools to improve reporting and communication across national and regional levels
Capacity-building	Continuous awareness and education promotion to combat vaccine misconceptions Incentives to reach vaccination targets Used community health care workers to bridge gap to communities	Inadequate information on disease epidemiology, treatment, and vaccines Lack of Internet connection at facilities to access and enter data systems	Create sustainable capacity building plans at regional and national levels Advocate vaccination to influential local and national leaders, religious leaders, and important members of society
Data management	Standardized data collection tools through registers and weekly reports Systematic collection of COVID-19 data from vaccination points to the national level through an online system Periodic data reporting, verification, and feedback mechanism to lower levels	Delays in data entry led to backlogs Lack of human resources to complete data entry	Build human capacity for data entry at vaccination points Integrate data collection systems to national online systems
Demand creation	Engaged and sensitized key stakeholders across village, ward, district, regional, and national levels Used peers and call back strategies to sensitize PLHIVs Aired local and national media coverage of vaccination uptake and dialogues One-on-one demand creation strategy effective at addressing myths	Types of vaccines affected acceptance rate Variance of COVID-19 vaccination acceptance among rural versus urban communities Competing family and community values	Maximize weekends to ensure no drop in vaccination progress Engage government-specific HIV organizations and religious leaders to reach PLHIV network Incorporate relevant media and press for widespread appeal and education
Supply chain	Single-dose vaccines accelerated vaccination coverage Weekly monitoring of vaccine inventory Stakeholders filled gap in transportation of vaccination distribution	Unable to meet demand for single dose vaccines Lack of daily vaccination monitoring at facility levels Vaccine wastage due to short half life	Consider client vaccination preference when forecasting demand Increase capacity for cold chain vaccination storage
Service delivery	Integrated COVID-19 vaccination activities at HIV clinics and community ART outreach services Merged vaccination activities with mass gatherings such as political events, religious gatherings, markets, sport outings, and sociocultural events Employed vaccine champions for hard-to-reach populations Political will and support from leaders at national and subnational levels	Lack of knowledge, misconceptions, and stigma around COVID-19 Logistic shortages including intermittent supply of vaccines, limited trained staff, lack of transport, small number of vaccination certificates Low commitment and buy-in among health care providers	Multifaceted approach of door-to-door, temporary fixed points, integration with other services, and community gatherings Improve logistic vaccination shortages to ensure consistent availability of supply Involve stakeholders at all levels to increase political will and vaccination support

Abbreviation: PLHIV, people living with HIV.

## Supplementary Material

GHSP-D-23-00281_supplement.pdf

## Notes

**Peer Reviewed**

**First published online:** May 23, 2024
