# Supplementary material for: Accelerating COVID-19 Vaccination Among People Living With HIV and Health Care Workers in Tanzania: A Case Study
Source: Glob Health Sci Pract. 2024 Jun 27;12(3):e2300281. doi: 10.9745/GHSP-D-23-00281 (PMC11216698; doi:10.9745/GHSP-D-23-00281)
Supplement: GHSP-D-23-00281_supplement.pdf [file GHSP-D-23-00281_supplement.pdf]

IMPROVING COVID-19 VACCINATION ENGAGEMENTS WITH HEALTH CARE WORKERS

AGENDA

Time	Topic
2:00 – 2:10	Welcome and Objectives
2:10 – 2:30	Vaccine Confidence Concepts
2:30 – 2:45	COVID-19 Vaccination Efforts in Tanzania
2:45 – 3:05	Building Vaccine Confidence and Demand among Healthcare Workers
3:05 – 3:15	Q&A/Break
3:15 – 3:50	How to Engage Unvaccinated People
3:50 – 5:00	Breakout Groups: Role Play Activity
5:00 – 5:20	Report Out
5:20 – 5:25	CDC Resources
5:25 – 5:30	Survey and Closing

SESSION OBJECTIVES

By the end of this training, you will be able to:

- Describe Tanzania's priorities for accelerating COVID-19 vaccine uptake
- Describe the basic facts about COVID-19 vaccines and key concepts related to vaccination confidence and demand
- Have effective conversations about vaccination with unvaccinated health workers
- Guide HCWs on how to promote the vaccine to patients and their families/households/networks
- Support effective vaccine deployment and promotion in health facilities

WHAT IS VACCINE CONFIDENCE?

DEFINING **VACCINE CONFIDENCE**

Vaccine confidence is the **trust** that patients, parents, or health providers have in:

- Recommended vaccines
- Providers who administer vaccines
- Processes and policies that lead to vaccine development, licensure, manufacturing, and recommendations for use

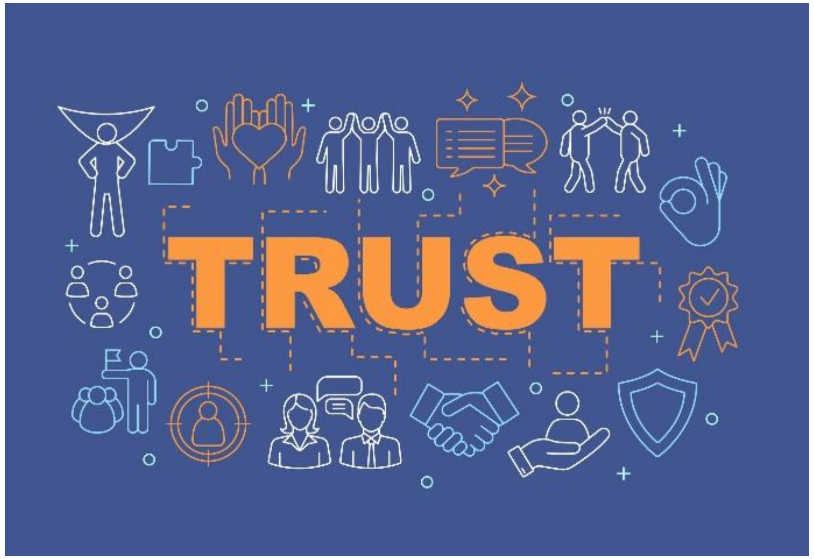

VACCINE CONFIDENCE IS **DYNAMIC**

Example Behaviors:

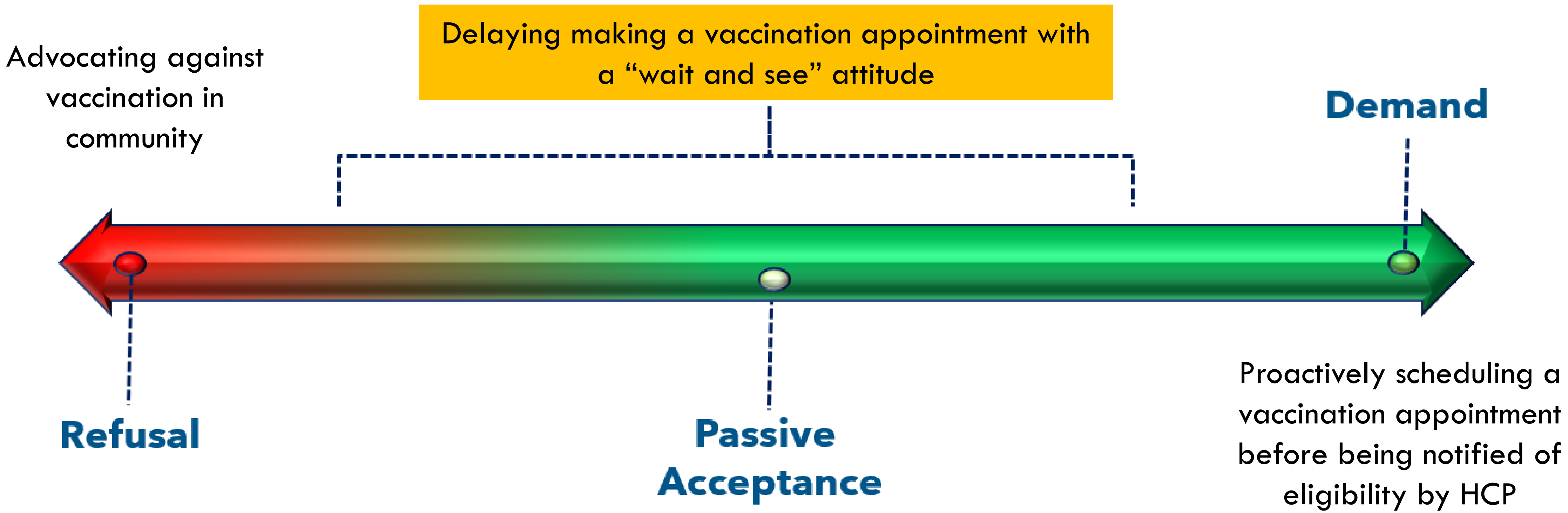

BUILDING **CONFIDENCE** AND GENERATING **DEMAND**

Vaccine Confidence

Building trust in the vaccine, the vaccinator, and the system

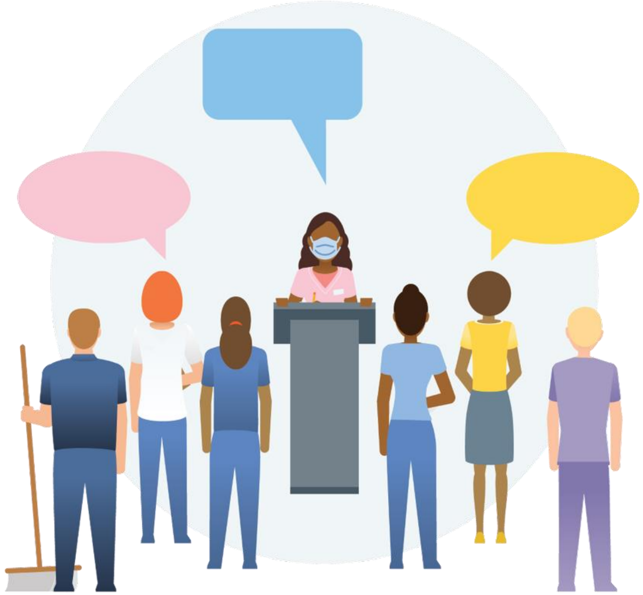

Vaccination Demand

Mobilizing individuals and communities to seek, support, and advocate for vaccines

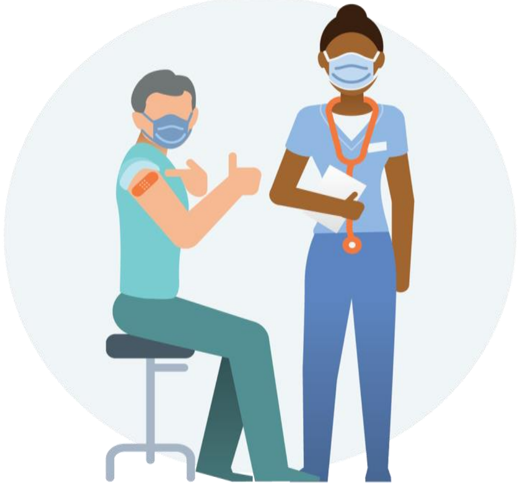

Ladder to Building Demand

Make vaccines:

- **Necessary** (indispensable for accessing things they want to get back to doing)
- **Normative** (presented as a social default)
- **Desirable** (appealing)
- **Convenient** (reduce out of pocket, social, and opportunity costs)
- **Beneficial** (health benefits outweigh risk of getting COVID-19 or perceived or real side effects from vaccination)
- **Accessible** (easy to get)

UNDERSTAND THE **VACCINATION JOURNEY**

- COVID-19 susceptibility & severity
- Vaccine safety & efficacy
- Vaccine recommendations

- Education about vaccine
- Explanation of what to expect/do (side effects)
- Questions answered

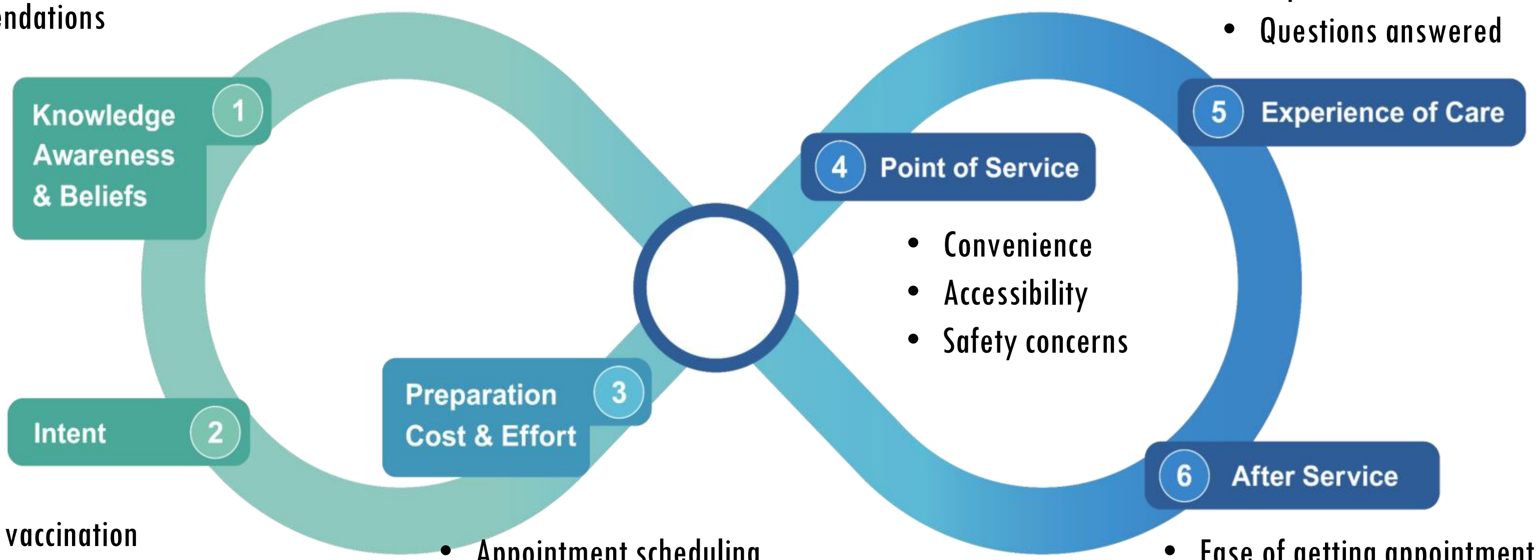

- Convenience
- Accessibility
- Safety concerns

- Trust in vaccination institutions
- Vaccination benefits
- Vaccination norms

- Appointment scheduling
- Vaccine/appointment costs
- Logistics to get to/from appointment
- Time spent at appointment

- Ease of getting appointment for second dose
- Adverse event monitoring
- Positive reinforcement

Modified from UNICEF Journey to Health, ESARO Network Meeting 2019

COVID-19 VACCINATION IN TANZANIA

TANZANIA'S VACCINATION ACCELERATION EFFORTS

- **COVID-19 vaccines are safe, effective, and widely available for free in Tanzania**
  - Vaccination remains the best tool in curbing the devastation of the COVID-19 pandemic and easing the burden on the healthcare delivery system to address other issues, including HIV/TB services

- **CDC Tanzania, through its implementing partners, is supporting the Government of Tanzania to accelerate uptake of COVID-19 vaccines**
  - Professional cadres (e.g., doctors, nurses, laboratorians and other clinicians)
  - Administrative, logistics, management, custodial, security staff

- **Being credible ambassadors for accelerating COVID-19 vaccine uptake**
  - Implementing partners' staff and contractors need to role-model the promoted behavior and get fully vaccinated

- **Intensification of targeted outreach and scaling up over time**
  - Targeted outreach to health care workers (HCWs) and community health workers (CHWs), people living with HIV (PLHIV) and the families/households/networks of PLHIVs

AVAILABILITY OF COVID-19 VACCINES IN TANZANIA

- COVID-19 vaccines are currently available in Tanzania to anyone age 18 years and above
- The vaccines are now available for free at nearly 7,000 sites across the country
- Government of Tanzania continues to rapidly expand vaccination sites all over the country
- Nearly one million people in Tanzania have received COVID-19 vaccines since August 2021
- Beyond Tanzania, more than 6 billion doses of COVID-19 vaccines have been given to billions of people around the world

STATISTICS ON COVID-19 VACCINE UPTAKE IN TANZANIA

Mainland (23rd - 29th October, 2021)	
Total number of vaccines distributed	1,023,400
New people vaccinated during the week	55,515
Cumulative number of people vaccinated	1,036,812
**Zanzibar (11th - 20th October, 2021)**	
Total vaccine doses received in Zanzibar	275,000
New people vaccinated this week	1,476*
Cumulative number of people vaccinated	35,231

*Data entry ongoing due to existence of backlog of forms at the vaccination centers

KEY THINGS TO KNOW ABOUT COVID-19 VACCINES

- COVID-19 vaccines are safe and available free of charge in Tanzania
- COVID-19 vaccines are effective at helping protect against severe disease and death from currently circulating variants of the virus that causes COVID-19
  - including the Delta variant
- COVID-19 vaccines were developed using science that has been around for decades
- As with all vaccines, you may have side effects after vaccination
  - These are normal and should go away in a few days
- Being fully vaccinated can help to protect your family, community, or elders who cannot get vaccinated
- Building defenses against COVID-19 is a community effort, everyone has an important role to play

BENEFITS OF COVID-19 VACCINATION

- COVID-19 vaccination is the best tool to help end the pain and suffering caused by the pandemic
- The vaccine prevents you from getting the disease, getting admitted to the hospital, and dying from COVID-19
- Get vaccinated regardless of whether you already had COVID-19. Evidence is emerging that people get better protection by being fully vaccinated compared with having had COVID-19
- When you get vaccinated you may also protect people around you, including your family members, other household members, and neighbors.
- If you don't get the vaccine and end up getting COVID-19, you also risk giving it to loved ones who may get very sick
  - Getting a COVID-19 vaccine is a safer choice.

BUILDING VACCINE CONFIDENCE AND DEMAND AMONG HEALTHCARE WORKERS

WHAT SHOULD HEALTH CARE WORKERS DO?

- **Get vaccinated** and become a Vaccination Champion
- **Initiate** vaccination dialogue with patients
  - Highlight vaccination benefits strongly
- **Facilitate** and support clients to get vaccinated
  - Show them where to get vaccinated
  - Escort them to the vaccination point if needed
- **Educate** clients about vaccine side-effects
  - Normal reactions to the vaccine versus serious adverse events
- **Follow-up** with clients who refuse vaccination
  - Phone call
  - Mobile outreach
  - Next visit

STRATEGIES FOR BUILDING VACCINE CONFIDENCE AMONG HEALTHCARE PERSONNEL

- Encourage senior leaders to be vaccine champions
- Host discussions where personnel at different levels can provide input and ask questions
- Share key messages with healthcare personnel through emails, breakroom posters, and other channels
- Talk with non-medical staff about the importance of getting vaccinated
- Encourage healthcare personnel to make their decision to get vaccinated visible
  - Record testimonial from health workers on why got vaccinated and provide medium where the videos can be shown at the health facility (e.g. in waiting area, during health talks, etc)

Q&A

HOW TO ENGAGE UNVACCINATED PEOPLE

WHAT IS MOTIVATIONAL INTERVIEWING?

- A collaborative communication style used to strengthen a person's own motivation and commitment to change.
- There is strong evidence for the impact of motivational interviewing in increasing vaccine confidence and uptake.
- Motivational interviewing is based on 3 main components:
  - Cultivating a culture of partnership and empathy
  - Fostering engagement in the relationship and targeting the goal of the intervention
  - Understanding the person and adapting to their specific needs

OPEN-ENDED QUESTIONS

Ask **open-ended questions** to encourage exploration of thoughts and feelings

Examples:

- *"How are you feeling about the vaccine?"*
- *"What concerns do you have?"*
- *"What reasons do you see to get the vaccine?"*

Remember: your goal is to helping the person move toward accepting the vaccine. Allowing them to talk is a big step!

AFFIRMATIONS

Use **affirmations** to acknowledge and build confidence in the person's innate capabilities

Examples:

- *"I can see that you really care about this."*
- *"It sounds like you have a lot of knowledge."*
- *"Thank you for being honest with me."*

REFLECTIVE LISTENING

Apply **reflective listening** to clarify your understanding and allow the person to hear their own words/thoughts/feelings reflected to them

Examples:

- *"What I hear you saying is…"*
- *"It sounds like you're feeling worried about…"*
- *"It sounds like you're unsure because…"*

SUMMARIZE

Summarize to let the person know they are being heard and to keep the discussion active and moving forward

Examples:

- *“You mentioned several things you would like to know more about…”*
- *“You said you would like to talk with your friends and family about…”*
- *“You mentioned you would like help with…”*

ASK PERMISSION, SHARE, AND VERIFY

Ask for **permission** to **share** information/advice and **verify** that the person has understood

Examples:

- *"May I share some information about the vaccine?"*
- *"Would it help if I shared about my own experience?"*
- *"Does this new information make sense?"*

MOTIVATIONAL INTERVIEWING TECHNIQUES

- Ask **open-ended questions** to encourage exploration of thoughts and feelings.
- Use **affirmations** to acknowledge and build confidence in the person's innate capabilities.
- Apply **reflective listening** to clarify your understanding and allow the person to hear their own words/thoughts/feelings reflected to them.
- **Summarize** to let the person know they are being heard and to keep the discussion active and moving forward.
- Ask for **permission** to **share** information/advice and **verify** that the person has understood.

LIMITATIONS OF MOTIVATIONAL INTERVIEWING

- Motivational interviewing skills require **training** and **experience.**
- The **time** required to establish relationships for MI is not always feasible.
- A motivational interview may not be **appropriate for every context.**
- People may **lack individual autonomy or freedom** to make changes.
- Motivational interviewing is **challenging for individuals who lack ambivalence and are highly confident** they do not wish to change.

MI CONCEPTS: FOR SUPPORTIVE INDIVIDUALS

- Strengthen their motivation and commitment to immunization by searching for opportunities to partner and collaborate with individuals while fostering a non-judgmental environment
- Provide information when requested and ensure it is clear to them
- Provide affirmations

Source: Dr. Arnaud Gagneur et al. Universite' de Sherbrooke. The EMMIE Program

MI CONCEPTS: FOR AMBIVALENT INDIVIDUALS

- Engage in reflective and empathetic (non-judgement) listening
- Show affirmation to emphasize their strengths
- Ask permission to provide information or to focus on a particular situation specific to the individual before providing information or discussing the situation or issue to foster partnership and collaboration
- Draw out ideas rather than imposing ideas (Evocation)
- Spark conversations on change talk by asking open-ended and double-reflective questions that is adapted to the vaccine hesitancy level or the individual
- Listen for change speech articulated by the individual

Source: Dr. Arnaud Gagneur et al. Universite' de Sherbrooke. The EMMIE Program

MI CONCEPTS: FOR VACCINE HESITANT INDIVIDUALS

- Use non-judgmental language to affirm that their resistance is a long-standing choice, adjust to their resistance level
- Show support for their autonomy to choose to or not to choose vaccination
- Express empathy rooted in complex reflections
- Ask open-ended questions to gauge what could be useful and helpful to them as they further reflect
- Draw out ideas rather than imposing ideas (Evocation)

Source: Dr. Arnaud Gagneur et al. Universite' de Sherbrooke. The EMMIE Program

MI CONCEPTS: FOR VACCINE HESITANT INDIVIDUALS CONTINUED…

- Create space for partnership and collaboration in lieu of providing new information to them.
- Ask open-ended questions on how they interpret the information and what they intend to do with the new information
- Highlight reasons for high level of vaccine hesitancy to create opportunities to exchange and share relevant information
- Do not address vaccine planning unless they explicitly request it.

Source: Dr. Arnaud Gagneur et al. Universite' de Sherbrooke. The EMMIE Program

REMEMBER…

- Be compassionate, show empathy, and be genuinely curious about the reasons why the person feels the way they do.
- Be sensitive to culture, family dynamics, and circumstances that may influence how people view vaccines.
- The goal is to understand, not to change their mind on the spot.
- These conversations take time and may continue over multiple visits and engagements.

RESPONDING TO QUESTIONS ABOUT VACCINES

If the person asks a question about vaccine safety or vaccine risks:

- ✓ Respond within the boundaries of your competence, ethics, and scope of practice.
- ✓ Respond with empathy and respect.
- ✓ Don't feel the need to over-explain things.
- ✓ Provide scientific information as needed but without going into confusing technical details.
- ✓ A simple response reassuring that the vaccine is **safe and effective** may be sufficient for some people.
- ✓ Refer the person to Government of Tanzania resources.

ROLE PLAY

- Open-ended questions
- Affirmation
- Reflective listening
- Summarize
- Permission, share, verify

- What did you notice about the conversation?
- What was done well?
- What could have been better?

MOTIVATION INTERVIEWING RECAP

- Health care workers (HCWs) are people just like everyone else.
- HCWs may have concerns about getting vaccinated or may have doubts about the vaccine.
  - Don’t shame them or discourage them from talking about their concerns, doubts, and questions
- Show **respect**, take time to **listen**, and help them **talk through their concerns.**
- Find opportunities to steer them towards talking about the **benefits of getting vaccinated.**
- A vaccinated HCW can become a powerful **Vaccination Champion.**

YOUR TURN!
BREAKOUT GROUP ROLE PLAY ACIVITY

BREAKOUT GROUP ROLE PLAY ACTIVITY

- Practice applying MI techniques in 3 separate scenarios.
- Each person will rotate between 3 roles in each group:
  - 1. Healthcare Provider
  - 2. Implementing Partner Staff
  - 3. Observers
- Instructions will be provided in secret to each role.
- Each scenario will last approximately 10 minutes.
  - Observers should consider the MI techniques we have reviewed.

MOTIVATIONAL INTERVIEWING TECHNIQUES

- Ask **open-ended questions** to encourage exploration of thoughts and feelings.
- Use **affirmations** to acknowledge and build confidence in the person's innate capabilities.
- Apply **reflective listening** to clarify your understanding and allow the person to hear their own words/thoughts/feelings reflected to them.
- **Summarize** to let the person know they are being heard and to keep the discussion active and moving forward.
- Ask for **permission** to **share** information/advice and **verify** that the person has understood.

ROLE PLAY

- Open-ended questions
- Affirmation
- Reflective listening
- Summarize
- Permission, share, verify

- What went well?
- What could have been improved?

REPORT OUT

- How did the conversation go?
- What went well?
- What was difficult?

HOW TO ENGAGE UNVACCINATED PEOPLE INCLUDING HEALTH WORKERS

- **Step 1**: Embrace an attitude of empathy and collaboration
- **Step 2**: Ask permission to discuss the COVID-19 vaccines
- **Step 3**: Use motivational interviewing to steer them toward accepting the vaccine
- **Step 4**: Respond to questions about the COVID-19 vaccines

CDC COMMUNICATION RESOURCES

COMMUNICATION RESOURCES

- Myths and Facts about COVID-19 Vaccines | CDC
- Talking with Patients about COVID-19 Vaccination | CDC
- Answering Patients' Questions about COVID-19 Vaccination | CDC
- How to Build Healthcare Personnel's Confidence in COVID-19 Vaccines | CDC

COMMUNICATION RESOURCES

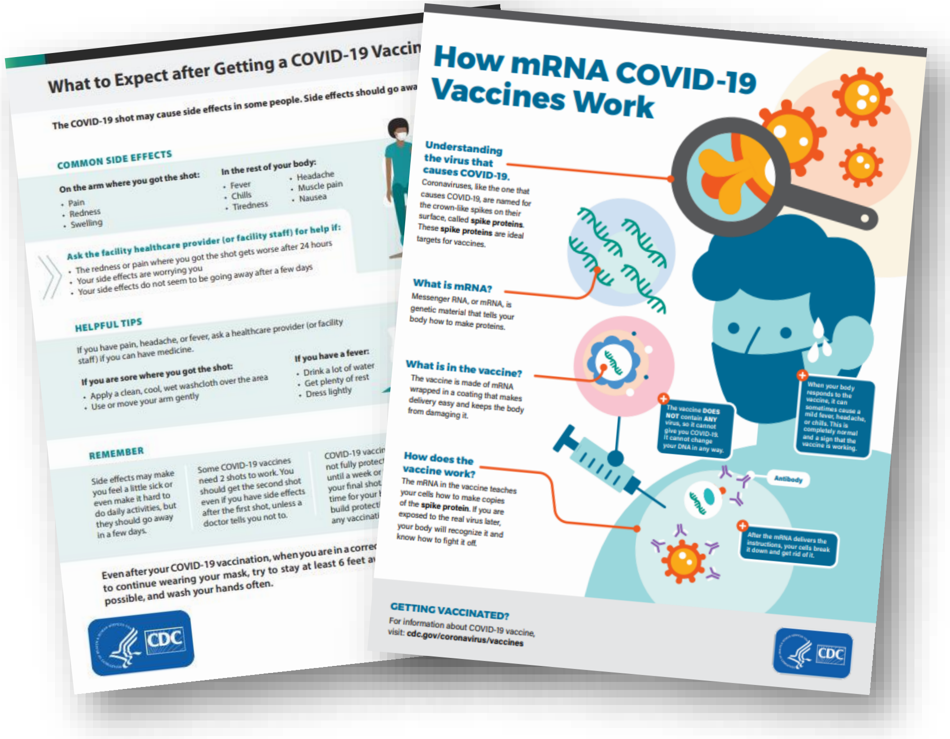

[Factsheets](#)

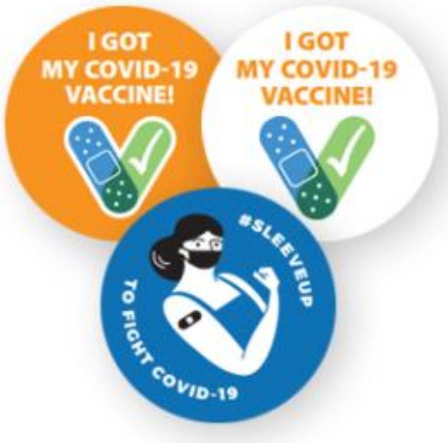

[Stickers](#)

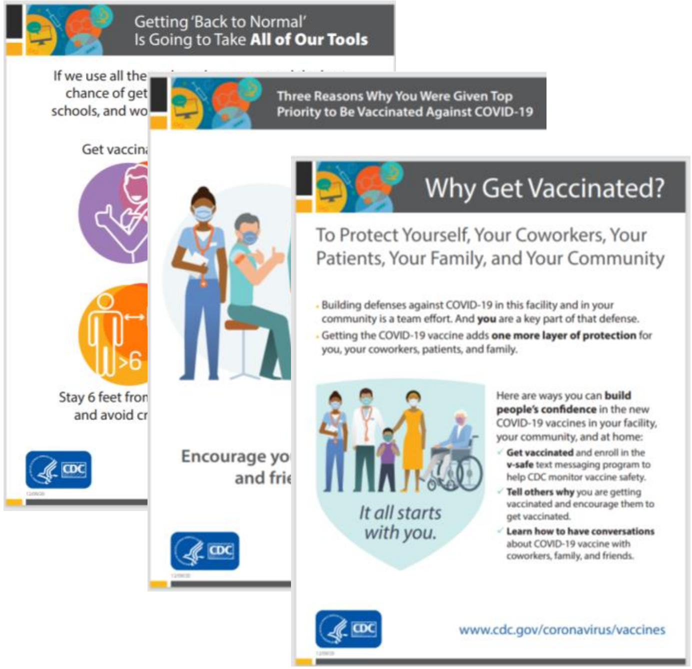

[Posters](#)

SURVEY + CLOSING

SURVEY

Thank you for your feedback!

THANK YOU!
